# The effect of air temperature on hospital admission of adults with community acquired pneumonia in Baotou, China

**DOI:** 10.1038/s41598-021-88783-7

**Published:** 2021-04-30

**Authors:** Wenfang Guo, Letai Yi, Peng Wang, Baojun Wang, Minhui Li

**Affiliations:** 1 Inner Mongolia Autonomous Region Academy of Traditional Medicine, No. 11 Jian Kang Street, Hohhot, 010020 Inner Mongolia China; 2 Inner Mongolia Hospital of Traditional Chinese Medicine, Hohhot, 010020 China; 3 The First Affiliated Hospital of Baotou Medical College, Baotou, 014000 China; 4 Baotou Central Hospital, Baotou, 014040 China; 5 Baotou Medical College, Baotou, 014060 China

**Keywords:** Climate sciences, Environmental sciences, Diseases

## Abstract

The relationship between air temperature and the hospital admission of adult patients with community-acquired pneumonia (CAP) was analyzed. The hospitalization data pertaining to adult CAP patients (age ≥ 18 years) in two tertiary comprehensive hospitals in Baotou, Inner Mongolia Autonomous Region, China from 2014 to 2018 and meteorological data there in the corresponding period were collected. The exposure–response relationship between the daily average temperature and the hospital admission of adult CAP patients was quantified by using a distributed lag non-linear model. A total of 4466 cases of adult patients with CAP were admitted. After eliminating some confounding factors such as relative humidity, wind speed, air pressure, long-term trend, and seasonal trend, a lower temperature was found to be associated with a higher risk of adult CAP. Compared to 21 °C, lower temperature range of 4 to –12 °C was associated with a greater number of CAP hospitalizations among those aged ≥ 65 years, and the highest relative risk (RR) was 2.80 (95% CI 1.15–6.80) at a temperature of − 10 °C. For those < 65 years, lower temperature was not related to CAP hospitalizations. Cumulative lag RRs of low temperature with CAP hospitalizations indicate that the risk associated with colder temperatures appeared at a lag of 0–7 days. For those ≥ 65 years, the cumulative RR of CAP hospitalizations over lagging days 0–5 was 1.89 (95% CI 1.01–3. 56). In brief, the lower temperature had age-specific effects on CAP hospitalizations in Baotou, China, especially among those aged ≥ 65 years.

## Introduction

Community-acquired pneumonia (CAP) refers to the infectious pulmonary parenchyma inflammation infection-acquired outside a hospital. CAP carriers also include those who are infected with the pathogen (of known latency) outside hospitals and develop symptoms in a hospital during the latent period^1^. CAP exists all over the world and threatens the health of the whole human race. It leads to approximately 1.6 million hospitalizations and 100,000 in-hospital deaths in the United States^2^. In Europe, about one percent of the population is infected with CAP, in which about 30–40% require hospitalization^3^. The estimated annual incidence of hospitalizations for CAP was 500–1000 per 100,000 persons in the Asia–Pacific region, but incidence in older adults is significantly higher^4^. A study showed that a total of 197,316 emergency hospital admissions for pneumonia with subjects aged ≥ 65 years accounted for 73.8%^5^. CAP is considered as the main infectious disease triggering sepsis or septic shock^6^ and the mortality rate among severe CAP patients in intensive care units (ICUs) can reach 49%^7–9^. The pathogens of CAP show significant change across different countries and regions and exhibit temporal variations as well^10,11^.

The air temperature, as a meteorological factor that is the most extensively distributed and the most influential, plays an important role in human survival. An extremely low or high temperature and abnormal changes beyond the tolerance of the human body have a negative influence on human health. These temperature conditions can directly promote or exacerbate respiratory diseases or indirectly increase the level of exposure to associated risk factors of respiratory tract diseases^12^. Researchers have assessed the possible effects of various meteorological factors on pneumonia and other respiratory diseases. Temperature, a well-known factor, is likely to be the least controversial^5,13–15^. Some researchers have reported J, U, or V-shaped relationships between temperature and CAP^15,16^ while the relationship patterns seem to vary by location^6–8^ and temperature range^17^, therefore, the effect of temperature on the hospital admission of CAP patients demonstrates a regional difference, thus resulting in different research results. Although the duration of cold periods in most areas will decrease with increasing global mean temperature, the effects of cold periods generally last for longer than that of a heat-wave^5^. Therefore, cold periods are still regarded as the main environmental threat to human health^5,18^. At present, research into the relationship between the hospital admission of adult CAP patients and air temperature is not frequently reported. Most research concentrates on childhood pneumonia or respiratory diseases and population-wide respiratory diseases^15,19^. Therefore, the purpose of the study is to evaluate the relationship between air temperature and the hospital admission of adult CAP patients.

## Methods

### Study region and data source

Baotou, located in the central region (109° 15′ E to 110° 26′ E; 40° 15′ N to 42° 43′ N) of Inner Mongolia Autonomous Region, China, is classified as a typical temperate zone with a semiarid continental monsoon climate, with four distinctive seasons. In order to determine the effect of temperature on hospitalization for CAP in different age groups, we referred to other studies^20,21^ and abstracted data on overall daily adult (≥ 18 years) CAP hospitalization admissions, including admissions of elderly subjects (≥ 65 years) and young adult pneumonia (< 65 years) from two tertiary comprehensive hospitals in Baotou. Based on the information systems of the two hospitals, the hospitalization data on CAP patients who are not younger than 18 years of age between 2014 and 2018 were screened; furthermore, the information pertaining to patient identification number, gender, age, admission time, diagnosis on admission, and international classification of diseases (ICD)-10 codes was derived. The ICD-10 codes for pneumonia ranged from J12 to J18.

The meteorological data in the corresponding period were obtained from Baotou Meteorological Bureau, and the data including daily average temperature, daily average relative humidity, daily average air pressure, and daily average wind speed were collected.

### Statistical analysis

The relationship between air temperature and the hospital admission of adult CAP patients in Baotou was studied by using a distributed lag non-linear model (DLNM). Moreover, the effects of various confounding factors (such as relative humidity, air pressure, wind speed, long-term trend, seasonal trend, weekday effect, and holiday effect) could be controlled. The established model is shown as follows:

$$ \begin{aligned} {\text{Log}}\left( {E\left( Y \right)} \right) & = \alpha + {\text{cb}}\left( {T,{\text{Lag}} = {14}} \right) + ns\left( {H,df = 3} \right) + ns\left( {W,df = 3} \right) \\ & \quad + ns\left( {P,df = 3} \right) + ns\left( {time,df = 4/year} \right) + \eta Holiday + \delta Dowt \\ \end{aligned}$$

where, *E*(*Y t*) denotes the expected hospital admission of adult CAP patients in the *t*th day; ɑ, cb, *ns*, lag, *df*, T, *H*, *W*, and *P* represent the intercept, crossed basis function, natural cubic spline function, the maximum lag time, the number of degrees of freedom, air temperature, relative humidity, wind speed, and air pressure, respectively. The parameters were incorporated into the model according to the non-linear effect, in which the maximum lag time was defined as 14 days and the other parameters were identified based on the Akaike information criterion (AIC); the number of degrees of freedom of temperature, humidity, wind speed, and air pressure were set to three; time denotes the time variable, with the number of degrees of freedom set to four per year; Dow and Holiday separately refer to the day of the week and legal holidays, which were introduced into the model by using the dummy variable method. The relationship between daily average temperature and the hospital admission of adult CAP patients was explored and the air temperature effect was described by using relative risk (RR). The reference temperature is the average summer temperature of 21 °C (June, July, August), because CAP hospitalization is lower in this three-month period than at other times. In these conditions, the risks of increasing the hospital admission of CAP patients at a low temperature under different lag times were separately calculated.

Through statistical analysis in R 3.6.2 software, the distributed lag non-linear model (DLNM) program package was applied in the DLNM model. The α-level was set to 0.05.

### Ethical approval

This study has obtained an ethical approval exemption from the Ethics Committee of Inner Mongolia Autonomous Region Academy of Traditional Medicine. As the hospital admissions data were statistical summary data and all analyzed data were anonymized, the Ethics Committee of Inner Mongolia Autonomous Region Academy of Traditional Medicine waived the need for patients to sign informed consent proformas allowing the use of research data. All methods were carried out in accordance with relevant guidelines and regulations.

## Results

Table 1 summarizes the hospital admission of adult CAP patients. A total of 4466 CAP patients were admitted to the chosen hospitals. The proportion of CAP hospitalizations in all respiratory hospitalizations showed that the proportion of patients aged ≥ 65 years (15.61%) was greater than that of patients aged < 65 years (9.87%).

**Table 1:** Descriptive summary of hospital admissions of adult CAP patients.

Admission data	Total (*n*)	Percentage of hospitalizations for CAP in all respiratory diseases (%)	Mean number of cases per day
< 65 years	1730	9.87	1
≥ 65 years	2736	15.61	2
Total	4466	25.48	3

*CAP* community-acquired pneumonia.

A descriptive summary of ambient temperature, relative humidity, and daily average wind speed data during the study period is provided in Table 2. During the research period, the highest air temperature in Baotou was 30.1 °C, with the average temperature of 8.2 °C, and an average air pressure, average humidity, and average wind speed of 902.2 Pa, 54.8%, and 2.9 m/s, respectively. Spearman correlation result indicates the correlation coefficient between CAP and meteorological factors in Table S1. Overall, there was no monotonic relationship between the daily CAP hospitalization and meteorological factors (*p* < 0.01). Therefore, other climate variables should be adjusted when assessing the effect of temperature on hospitalizations for CAP.

**Table 2:** Descriptive summary of climate variables in Baotou during 2014–2018.

Variables	Min	P5	P25	P50	P75	P95	Max	Mean (SD)
Mean temperature (°C)	− 19.7	− 11.4	− 3.6	10.1	19.6	25.0	30.1	8.3 (12.5)
Relative humidity (%)	11.5	29.0	43.0	55.3	66.0	81.0	97.0	54.8 (15.8)
Pressure (PA)	885	892	896.6	902.2	907.1	913.5	924.7	902.2 (6.7)
Wind speed (m/s)	0.9	1.4	2	2.7	3.6	5.2	10.1	2.9 (1.2)

*Min.* minimum, *P5* 5th percentile, *P25* 25th percentile, *P50* 50th percentile (median), *P75* 75th percentile, *P95* 95th percentile, *Max.* maximum, *SD* standard deviation.

**Figure 1:**
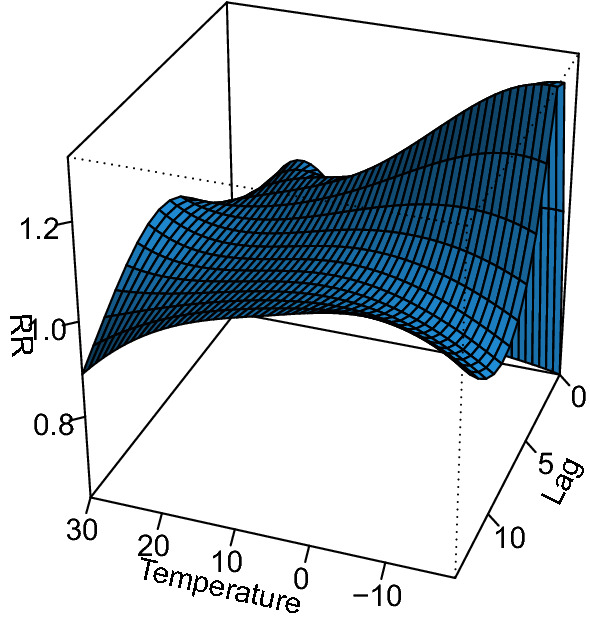
3-d diagrams of the relationship between air temperature and the hospital admission of adult CAP patients.

Figure 1 shows the RR of hospital admission for CAP associated with temperature using 21 °C as reference value. The effect of temperature on CAP hospital admissions was non-linear, and the RR was increased at lower temperature and with the highest risk at a temperature of -10 °C.

**Figure 2:**
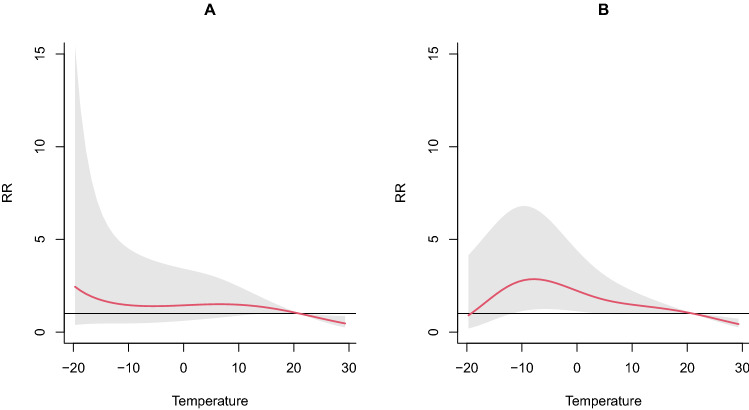
Cumulative effects for exposure–response relationship between temperature and CAP ((**A**): < 65 years; (**B**): ≥ 65 years). Note: the hatched area represents the 95% confidence interval.

Figure 2 shows the cumulative effects in terms of exposure–response between temperature and CAP hospitalizations. It appears an N-shaped pattern for those aged ≥ 65 years. The risk of hospital admission increases as the air temperature falls from 21 to − 11 °C but then it starts to decline with the highest risk of 2.80 (95% CI 1.15–6.80) at a temperature of − 10 °C (Fig. 2B). For those < 65 years, a lower temperature was not associated with the number of CAP hospitalizations.

**Figure 3:**
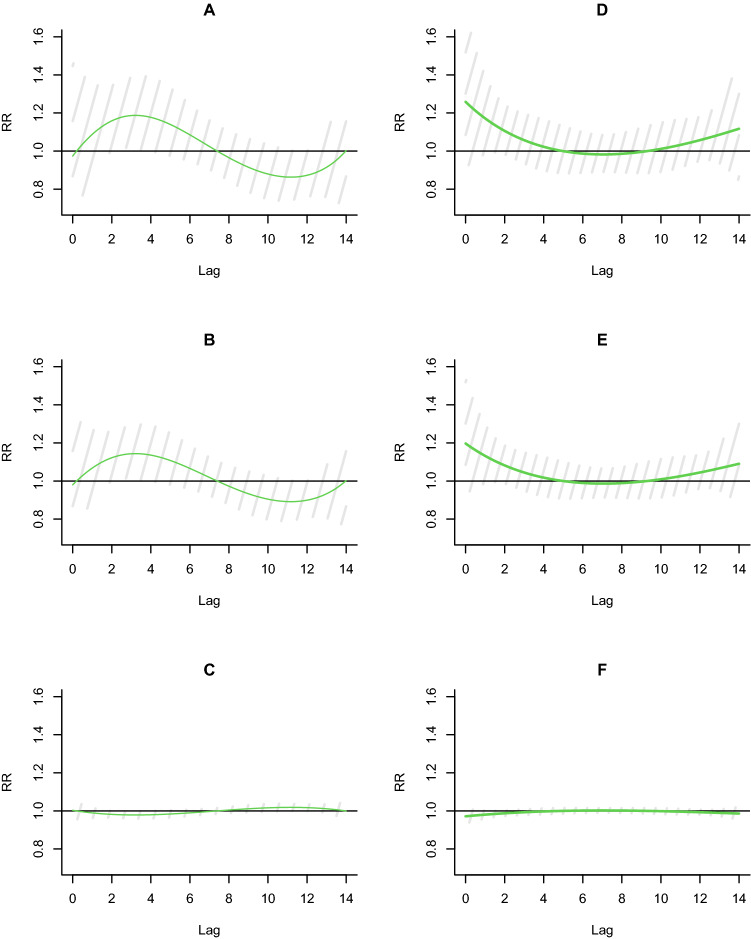
Lag effects of hospitalization risk of pneumonia exposure to low temperature and high temperature (≥ 65 years: (**A**-**C**) refer to the P5, P25, and P95 percentiles of daily average temperature, which are − 11.4 °C, − 3.6 °C, and 25 °C, respectively; < 65 years: (**D**-**F**) refer to the P5, P25, and P95 percentiles of daily average temperature, which are − 11.4 °C, − 3.6 °C, and 25 °C, respectively).

Taking 21 °C as a reference, the lag effects of CAP hospitalization risk after exposure to low temperature (P5: − 11.4 °C, P25: − 3.6 °C) and high temperature (P95: 25 °C) are illustrated in Fig. 3. The results showed that the duration of pneumonia hospitalization was shorter and RR values were higher at a lag of 2–4 days. Then a trend towards fewer hospital admissions after a lag of 7 days appears while the lag effect was not statistically significant among those aged ≥ 65 years. The high temperature had no lag effects on CAP hospitalizations.

Cumulative lag RRs of low temperature (P5, P25) with CAP hospitalizations by different days are displayed in Table 3. For those aged ≥ 65 years, the cumulative RR of CAP hospitalizations with lower temperature (P5) over lag days 0–5 was 1.89 (95% CI 1.01–3.56).

**Table 3:** Cumulative RRs of lower temperature with CAP hospitalizations by different lag days.

Group	Temperature (℃)	Lag of 0–5 days	Lag of 0–7 days	Lag of 0–14 days
< 65 years	− 11	2.17 (0.97–4.83)	1.94 (0.82–4.64)	1.24 (0.43–3.56)
	− 4	1.83 (0.98–3.42)	1.68 (0.85–3.32)	1.19 (0.52–2.69)
≥ 65 years	− 11	**1.89 (1.01**–**3.56)**	1.72 (0.87–3.41)	1.94 (0.85–4.44)
	− 4	**1.65 (1.01**–**2.70)**	1.53 (0.89–2.61)	1.68 (0.88–3.20)

Bold estimates are statistically significant.

## Discussion

The results showed that older adult (age ≥ 65 years) CAP hospitalizations in Baotou is more likely to be influenced by a low temperature and most of the hospital admissions were attributed to moderately cold weather. The trends differ from those observed in other studies^15,16^. Based on a study carried out in Shanghai, China, there is a V-shaped relationship between the daily average temperature and the rate of hospitalization of CAP patients, showing an optimum temperature of 18°C^15^. In a study conducted in Beijing^16^, the effects of warm periods (from April to September) and cold periods (from October to March in the following year) were separately discussed and a J-shaped exposure–response function relationship between the average temperature and mortality was investigated. In our study, the one explanation for the overwhelming majority of the attributable risk occurring in the moderate cold temperature range may be that moderate cold weather includes most of the days during the study and extreme cold weather is rare. In addition, it was marked on the basis of seasonal representatives of frequent dust-storms in April, frequent precipitation in September, and winter heating beginning from November in Baotou City^22^. Epidemiological studies have demonstrated the exacerbation of respiratory diseases following sandstorm-derived particulate matter exposure and winter heating^22,23^. This could increase CAP hospitalizations during moderate cold weather. This finding can also be contextualized within people’s health behavior: when the temperature changes from warm to slightly cold, people are still used to dress lightly for a period of time while when they gradually feel the sharp temperature change, they begin to wear more clothes. The other reasons for the unidirectional risk effect of a moderate cold temperature may be attributed to differences in the pathogen spectrum, prevailing geographical environment, and research methods.

The results also showed that people aged ≥ 65 years were more susceptible to a low temperature in terms of pneumonia-induced hospitalization. The result is similar to that from a study performed in Texas, USA and it is found that a low temperature is implicated in the growth of the incidence risk of CAP among elderly populations^18^. Temperature shows dual effects on diseases: on the one hand, it affects the physiological function and immune functions of the organism; on the other hand, it influences the spread of respiratory viruses^24,25^. When cold air is inhaled together with higher-pressure air, it stimulates the respiratory tract, thus causing muscle spasm, mucociliary movement decreased and further changes in membrane permeability increased the frequency of pneumonia^20,26^. The elderly population generally also has a lower thermoregulatory capacity and relatively weak immunological defenses, which increase their vulnerability to respiratory tract infection due to cold weather^5,12,27^. Moreover, due to their generally poorer health, the elderly show a high comorbidity, which will strengthen their susceptibility^9,28^. Additionally, several common respiratory viruses are implicated in the etiology of pneumonia in older adults, such as influenza, respiratory syncytial virus (RSV), hMPV, and coronaviruses. The influenza virus is the viral pathogen that is most well recognized in older adults and is the cause of significant morbidity and mortality in this age group^29^. The epidemic activities of RSV and invasive pneumococcal disease are negatively correlated with temperature^30,31^. The lower temperature is conducive to the propagation of influenza viruses while many hospitalized CAP patients are infected with influenza^32^. Furthermore, in the cold season, people will choose to turn on the heating and some areas heated by coal will suffer increased pollution which could increase the chance of pneumonia infection^22,33^. When it is cold, people spend more time indoors and therefore are closer together which could increase the risk of contracting an airway pathogen^34,35^.

Our study found that cold temperatures had a lag of 2–7 days for hospitalization for CAP, after 7 days, the effect was not statistically significant, which was similar to previous research results^15,20,36^. One reason for this is that microorganisms have an incubation period. The incubation periods of several common respiratory viruses usually are between 1 and 8 days^28^ such as influenza, RSV, hMPV, and coronaviruses. In addition, the elderly with a weaker immune system tend to develop clinical symptoms of pneumonia earlier than younger people when exposed to low temperatures, and the disease is more severe and usually leads to timeous hospitalization^10,34^. However, in other regions of China, the lag effects of meteorological factors were found to be 14 days or longer^33,37^, which may be caused by differences in climate, population susceptibility, and medical conditions.

This study has some limitations: first, this is an ecological study using population-level data, individual risk factors, comorbidities, and lifestyle, could not be included; therefore, our results should be considered as hypothesis-generating rather than confirmative. Second, we did not have data on outdoor air pollution, which is independently associated with CAP hospital admission and may modify the relationship between temperature and CAP. Third, we have not acquired clinical information such as chest X-ray, CT scan, or ultrasound scan data on the patients and rely solely on ICD-10 discharge diagnosis of pneumonia, therefore the possibility that some of the patients may have been misclassified cannot be excluded although we have excluded their pneumonia in hospital (HAP) or at the ICU (VAP) according to the diagnosis on admission. Fourth, the available monitoring data pertinent to outdoor meteorological factors taken from the monitoring stations were applied, instead of measured data relating to the exposure of individuals. This possibly leads to error in exposure classification data. Furthermore, only hospitalized CAP patients were explored while outpatients were not covered, therefore, the effects of meteorological factors on hospitalized CAP patients observed in the present study are possibly not applicable to the overall population of CAP patients. Therefore, it is deemed necessary to generalize the research results to other geographical areas, especially those with different climates and prevailing weather regimes.

## Conclusion

This study adds to the growing body of information on ambient temperature and hospital admission of adults with CAP. A lower temperature was associated with a higher risk of CAP, especially in the group aged ≥ 65 years. Furthermore, colder temperatures had a lag of 0–7 days for CAP hospitalizations.

## Supplementary Information

Supplementary Information.
