# Supplementary material for: The effect of air temperature on hospital admission of adults with community acquired pneumonia in Baotou, China
Source: Sci Rep. 2021 Apr 30;11:9353. doi: 10.1038/s41598-021-88783-7 (PMC8087821; doi:10.1038/s41598-021-88783-7)
Supplement: Supplementary file 1 — Supplementary Information. [file 41598_2021_88783_MOESM1_ESM.docx]

**The effect of air temperature on hospital admission of adults with** **community acquired pneumonia in Baotou, China**

Wenfang Guo, Letai Yi, Peng Wang, Baojun Wang, Minhui Li

Table S1. Spearman correlation among meteorological factors

| Variables | T | P | H | W |
| --- | --- | --- | --- | --- |
| T | 1 |  |  |  |
| P | -.817** | 1 |  |  |
| H | 0.002 | 0.031 | 1 |  |
| W | .104** | -.112** | -.166** | 1 |
| Note: *<0.05, **<0.01. T: Mean temperature; H: Relative humidity; P: Air pressure; W: Wind speed | | | | |
